# Ovarian cancer: Current status and strategies for improving therapeutic outcomes

**DOI:** 10.1002/cam4.2560

**Published:** 2019-09-27

**Authors:** Ashwin Chandra, Cima Pius, Madiha Nabeel, Maya Nair, Jamboor K. Vishwanatha, Sarfraz Ahmad, Riyaz Basha

**Affiliations:** ^1^ Texas College of Osteopathic Medicine UNT Health Science Center Fort Worth TX USA; ^2^ Miami Medical School Miami FL USA; ^3^ Graduate School of Biomedical Sciences UNT Health Science Center Fort Worth TX USA; ^4^ AdventHealth Cancer Institute Orlando FL USA

**Keywords:** biomarkers, clinical trials, ovarian cancer, ovarian cancer screening

## Abstract

Of all the gynecologic tumors, ovarian cancer (OC) is known to be the deadliest. Advanced‐stages of OC are linked with high morbidity and low survival rates despite the immense amount of research in the field. Shortage of promising screening tools for early‐stage detection is one of the major challenges linked with the poor survival rate for patients with OC. In OC, therapeutic management is used with multidisciplinary approaches that includes debulking surgery, chemotherapy, and (rarely) radiotherapy. Recently, there is an increasing interest in using immunomodulation for treating OC. Relapse rates are high in this malignancy and averages around every 2‐years. Further treatments after the relapse are more intense, increasing the toxicity, resistance to chemotherapy drugs, and financial burden to patients with poor quality‐of‐life. A procedure that has been studied to help reduce the morbidity rate involves pre‐sensitizing cancer cells with standard therapy in order to produce optimal results with minimum dosage. Utilizing such an approach, platinum‐based agents are effective due to their increased response to platinum‐based chemotherapy in relapsed cases. These chemo‐drugs also help address the issue of drug resistance. After conducting an extensive search with available literature and the resources for clinical trials, information is precisely documented on current research, biomarkers, options for treatment and clinical trials. Several schemes for enhancing the therapeutic responses for OC are discussed systematically in this review with an attempt in summarizing the recent developments in this exciting field of translational/clinical research.

## Graphical abstract

This review article provides an in‐depth information on current treatment strategies and available clinical trials for ovarian cancer. Systematic review of strategies for enhancing the therapeutic response in ovarian carcinoma summarizing the recent developments in translational and clinical research.

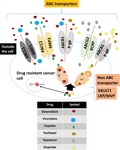

## 1 INTRODUCTION

Ovarian cancer (OC) is the deadliest cancer among women placing it with 4th place for all the fatal disease among women. Cancer statistics from 2019 show that the estimated number of new cases is 22 240 with deaths around 14 170 cases.^1^ There are three histological types associated with the disease. The most common is epithelial OC (EOC). Patients with this fatal disease have only 45.6% 5‐year survival rate.^2^ The survival rate in general increases up to 70% if effective early stage detection is possible. Early‐stage detection rate for this disease is as low as 20%. For most of the patients the late stage detection with advanced stage of cancer leads to low survival rate of 35%. In the case of recurrent EOC there is no satisfactory cure till to date.

Several aspects influence the progression of the disease. Genetic and epigenetic factors are the most important ones among them. Nearly 10%‐15% of familial OCs result from breast cancer gene mutations BRCA1 and BRCA2.^3^ The characteristic feature of these cancers is that they are multifocal and progress rather quickly. Mutations and the loss of the TP53 function are found in 60%‐80% of the familial and sporadic cases of the disease.^3^ These oncogenes will turn on different signaling pathways that leads to pathogenicity. Higher rate of thrombosis associated with OC is due to such activation of coagulation pathways by OC.^4^, ^5^

## 2 OVARIAN CARCINOMA‐PATHOBIOLOGY

Ovarian carcinoma is heterogeneous in nature. The disease progresses through several molecular level changes. Mainly there are three areas in the ovary where the tumor is developed. Surface epithelium is where majority of the malignancy is developed from. It is presented in different type of histology. Serous ovarian carcinoma (SOC) is the most common one and is presented at old age. Endometrioid carcinoma is presented at young age and associated with endometriosis. Mucinous carcinoma and clear cell carcinoma are also presented at young age. The other areas where the OC is developed are the germ cells and stroma.

The complexity in these malignancies arises from the microenvironment affected by changes in genetic factors. The degree of complexity varies according to the changes in epigenetic factors too. Understanding the tumor microenvironment is key for its diagnosis, treatment options, and survival. The microenvironment varies for different type of ovarian carcinomas with changes in gene expression leads to different tumor markers. The tumor markers play crucial role for the development of targeted therapies.^3^ Abnormal expression of homeobox (HOX) has been shown in histologic types developed at the embryonic stage.^6^ HOXA9 is absent in normal ovarian cells. High‐level expression of HOXA9 in SOC is found at the embryonic stage during fallopian tube formation. Abnormal level of HOXA10 is linked to endometroid carcinoma and HOXA11is linked to mucinous carcinoma.^6^ As far as the treatment goes, platinum‐ and taxane‐based chemotherapy have been shown to be successful for serous and endometrioid cancers, compared with clear‐cell cancers and mucinous histology type cancers.^3^

## 3 OC SCREENING TESTS

Low endurance rate for OC patients is because of the late‐stage detection and diagnosis of the disease. Early stage detection and diagnostic tools for screening OC are not efficient. Research is in progress for developing efficient diagnostic processes for OC. Transvaginal ultrasound (TVUS) is one of the current screening process for OC. Blood test for CA125, a tumor marker for OC is another common screening test for OC.^7^ Transvaginal ultrasound will identify the growth and masses in the scanned area. It will not differentiate between the malignant and benign masses. CA125 is elevated in ovarian carcinoma. But it is not specific to OC, hence a combination of TVUS in patients with high levels of CA125 can be a better screening tool for OC diagnosis.^8^

## 4 BIOMARKERS FOR OC

As the disease progresses, it gets even harder to treat and manage the patients. Only 20% of those affected cases have an early detection of the ailment. Many healthcare professionals confused OC with other urologic, abdominal, and gynecologic diseases because of the overlap in signs and symptoms, resulting in late detections. Ovaries do not have a peritoneal covering; therefore, the cancer spreads locally to the peritoneal cavity, resulting in symptoms. Absence of effective testing tools and equipments further delay the detection process for OC.^9^ As noted earlier, the early detection is crucial in increasing survival rates for advanced‐stage OC patients. Biomarkers are divided into diagnostic, prognostic, predictive, and response categories. Poor sensitivity and lack of specificity are the challenges for majority of biomarkes that have been studied. Although, the common biomarkers currently used are CA125, Human Epididymis Protein 4 (HE4), and mesothelin,^9^, ^10^ and their use in combination is often feasible.

### 4.1 CA125

Currently, the disease progression and treatment efficacy in OC patients is monitored using TVUS and elevated CA125 expression.^11^, ^12^ Elevated CA125 levels are present in about 80% of advance stage OC patients. For early stage OC patients elevated CA125 level is present in 50% only.^13^ New studies from Jennings group demonstrated the association of Neu5Gc‐glycans and SubB2M for detecting CA125 and using as an effective tool for the diagnosis and outcomes in stage II and IV patients.^14^

### 4.2 HE4

This is another maker for OC. Elevated HE4 expression is present in OC patients compared with normal and other nonmalignant diseases for women.^15^, ^16^ HE4 and CA125 are the biomarkers used in a study of women (n = 531) who has pelvic masses. 93.8% of these women were predicted for high‐risk ovarian carcinoma.^15^ In the US, HE4 is only approved as maker for OC for disease recurrence or progression.

### 4.3 Mesothelin

A 40‐kD protein associated with cell survival, tumor progression, and adherence. It is present in normal mesothelial cells. Increased levels of mesothelin is presented in blood samples of 40%‐67% of patients with OC.^16^, ^17^, ^18^, ^19^ The high expression level of mesothelin in OC identifies it as strong candidate for targeted therapy.

### 4.4 OVA1

A multiple biomarker‐based test OVA1 (Ovarian Malignancy Algorithm) is currently used for the evaluation of risk level of OC patients.^20^ Microglobulin Beta2, CA125, transthyretin (pre‐albumin), ApoA1, and transferrin are the biomarkers in OVA1. OVA1 analyze serum levels of these biomarkers. The OVA1 algorithm combines the results of these levels with information on the menopausal status of the patient for OC risk group classification.

### 4.5 DOvEEgene

It is an ongoing clinical trial (NCT02288676) study sponsored by McGill University, Canada. In advanced OC, treatment efficiency was studied using computed tomography (CT) perfusion.^21^

Numerous studies, and trials involving OC biomarkers that are being conducted across the globe. A summary of the ongoing biomarker studies in OC detection is given in Table S1 (Source: http://Clinicaltrial.gov).

## 5 OC TREATMENT STRATEGIES

The treatment strategies for different type of cancer depends on its pathological stages. Early detection will help to have the treatment options that are promising and effective. Current treatment options are combining debulking surgery and drug treatment and radiation therapy. Some of the advanced level treatment options include targeted therapy, immunotherapy, and hormone therapy. Chemotherapy is the most vital part of OC treatment. Chemotherapeutic agents can be administered via intravenously (IV), intraperitonially (IP), or by IV/IP combination. In neo adjuvant treatment plan chemotherapy was done before the surgery. IP/IV combination delivery of chemotherapeutic agents is the preferred mode drug administration for OC patients with cytoreduced disease.^22^, ^23^, ^24^ Treatment of peritoneal area is most effective when the chemotherapeutic agents are administered via IP route.^25^ Compared with the IV carboplatin chemotherapy, the IP carboplatin chemotherapy is well tolerated in advance stage OC patients undergoing surgery followed by neoadjuvant.^26^

Chemotherapeutic agents will be selected for treatment based on the stage of OC. Platinum containing drugs (cisplatin and carboplatin) and taxane family (paclitaxel and docetaxel) are frequently used chemotherapeutic agents for treatment of OC.^27^ Carboplatin is the preferred choice over cisplatin due to its reduced toxicity, and side effects with equivalent response rate and survival outcomes.^28^, ^29^, ^30^ Sensitivity of the chemotherapeutic agent is important during the drug selection process of OC. Gemcitabine, doxorubicin, and bevacizumab are the drugs used for treatment for cisplatin and carboplatin‐resistant ovarian carcinoma.^31^, ^32^, ^33^ Usage of high‐dose chemotherapeutic agent will lead to complications due to side effects and can result in termination of treatment plan. Since the OC cells undergo molecular level changes over the time and may lead to resistance to chemotherapy. A list of currently approved chemotherapeutic agents for OC therapy and their mechanism(s) for anti‐cancer activity is summarized in Table S2.

## 6 ROLE OF APOPTOTIC GENES AND TARGETED THERAPY

The biological phenomena by which the body gets rid of unnecessary cells in order to maintain homeostasis is known as apoptosis. OC, among others, has several genes working against apoptosis, which allows cancerous cells to flourish instead of being killed off. Candidates involved in both intrinsic and extrinsic pathways were studied. Bcl‐2 family proteins and Tyrosine‐protein kinases, respectively, facilitates intrinsic and extrinsic apoptosis, while inhibitor of apoptosis (IAP) proteins are associated with both intrinsic and extrinsic pathways. Bcl2 is anti‐apoptotic 34 and is expressed in high concentration in OC. 35, 36 Additionally, Bcl2 modulates resistance to chemotherapy and decreases survival, along with Bcl‐X and Mcl‐1 in OC patients.^36^, ^37^, ^38^ Conversely, Bid, Bad, Bax, and Kak all respond to the treatment by inducing apoptosis 39 and improve the survival. Clinical trials for treatment with Bcl‐2 inhibitors improved the response to cisplatin, and this has also been seen in preclinical models of OC studies.^39^, ^40^

Another anti‐apoptotic gene family is the IAP proteins. Survivin is a well characterized inhibitor for apoptotic proteins present in ovarian and other type of cancer cells.^41^ Survivin plays a significant role in cell division and thus control apoptosis. Animal studies have shown that targeting survivin with suppressor drugs resulted in tumor growth suppression and enhanced sensitivity to chemotherapeutic agents.^41^

Tyrosine‐protein kinase Met (c‐Met) linked to poor treatment outcomes for cancer chemotherapy is upregulated in OC.^42^, ^43^, ^44^, ^45^ Increased levels of c‐Met impacts cell proliferation, infiltration, angiogenesis, and endurance.^46^, ^47^, ^48^ Antiapoptotic activity of c‐Met linked to chemo resistance for therapies.^46^ Radiotherapy induces c‐Met expression and triggers the series of signals that increases the pro‐survival process and spreads the response of treatments.^49^ An in vitro study has shown that by treating OC cells with c‐Met inhibitors, cell proliferation has been significantly reduced and increased apoptosis of cancer cells was observed.^50^

**Figure 1:**
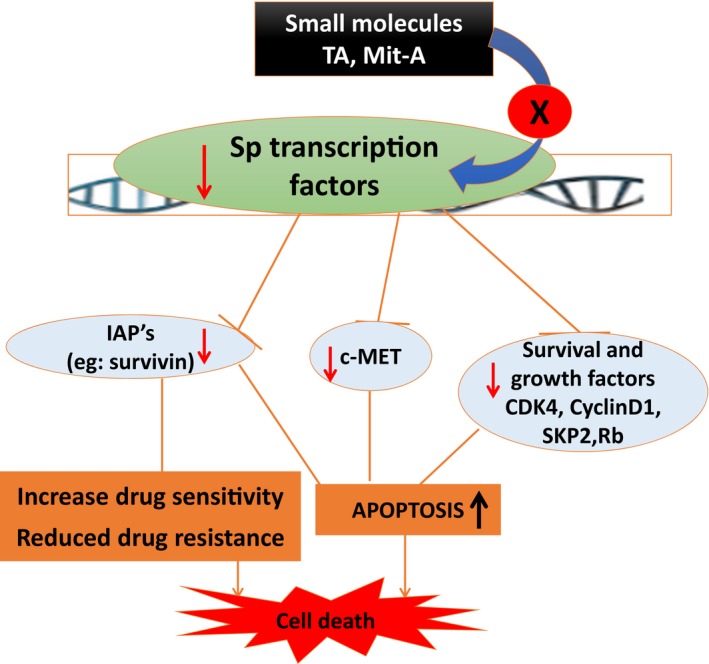
Association of Sp transcription factors in anti‐cancer activity. Small molecules like tolfenamic acid (TA), Mithramycin A are shown to inhibit specificity protein (Sp) family of transcription factors and will result in increased apoptosis of cancer cells

A class of transcription factors known as specific proteins (Sp) regulates VEGF (vascular endothelial growth factor) expression with functional variation. Thus, Sp transcription factors have crucial impact on tumor expansion and metastasis.^51^ Association of Sp transcription factors in anti‐cancer activity is illustrated in Figure 1.

## 7 MULTIPLE DRUG RESISTANCE

Research is in progress for understanding the mechanism of drug resistance in OC. Some of these mechanisms include increased DNA repair, overexpression of surface p 170‐glycoprotein, increased cellular levels of glutathione (GSH) and glutathione *S*‐transferases causing de‐toxification of platinum agents and taxol.^52^ Cancer cells develop certain transport proteins that help them to eliminate the effective dosages of drugs from the cells causing multiple drug resistance (MDR).^53^, ^54^

**Figure 2:**
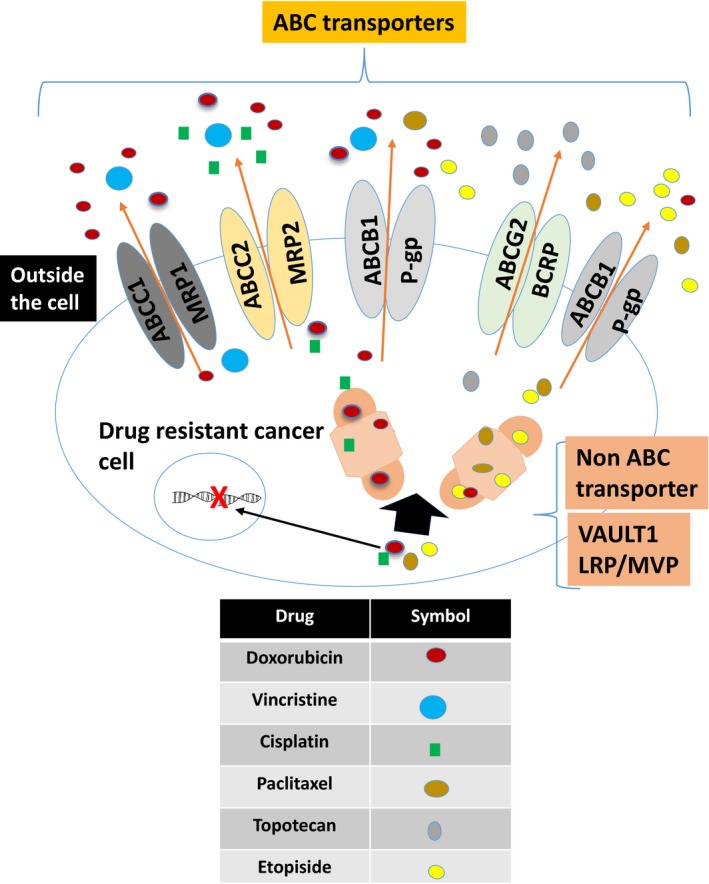
Dug resistance mechanisms: A schematic representation of proteins involved in drug resistance mechanism of commonly used chemotherapeutic agents for ovarian cancer chemotherapy. The classical multiple drug resistance produced by ABC transporters and non‐ABC transporters are illustrated

The “Classical” MDR is resulted from higher level of MDR‐1 gene that code for 170‐kD ATP‐dependent glycoprotein Pgp. Pgp causes reduction of cellular levels of cytotoxic drugs within the cells by transporting the drug outside the cells against the concentration gradient. Chemotherapy often upregulates the expression of P‐gp on cancer cells resulting in MDR. Resistance to multiple drugs is associated with P‐gp overexpression and includes paclitaxel, vincristine, and doxorubicin.^55^, ^56^, ^57^, ^58^ Cells that do not express P‐gp acquire other methods for drug resistance. Amplification of MDR‐associated protein gene (MRP) has been found in such cells that encodes a protein MRP, which expelling the drug out of the cells.^58^, ^59^ The MRP1 coded by ABCC1 and MRP2 coded by ABCC2 genes. They induce resistance to many cancer drugs, especially with the widely used cisplatin in OC.^55^, ^56^, ^57^, ^58^ A schematic representation of the proteins involved in drug resistance mechanism of commonly used chemotherapeutic agents for OC is given in Figure 2.

In yeast and mammalian system, copper transporter CTR1 is one of the transporters mediating uptake of platinum compounds.^60^ Cisplatin reduces its cellular influx by rapid degradation of CTR1 resulting in drug resistance.^61^, ^62^ Increased CTR1 expression by the cells results in an increased platinum concentration and decreased resistance to cisplatin.^62^ Combination of platinum drugs with bortezomib, a modulator for copper transporter expression, is a current option for platinum‐resistant solid tumors.^63^

Several epigenetic changes have been observed in cisplatin‐resistant human cells that open new avenues to study drug resistance among the OC cells. It has been observed that certain individual cells among the cancer cell population attain the reversible state of drug tolerance to prevent the eradication of the population by potential lethal exposure.^64^ Altering the chromatin state and engaging IGF‐1, insulin‐like growth factor will increase drug resistance. Treating with Inhibitors of IGF‐1 receptor will reverse this process and can be an important therapeutic strategy. In addition, by inactivating the cytotoxic genes like folate binding gene (FBP) in cancer cells, DNA hypermethylation plays critical role(s) in generating the multiple drug‐resistant phenotypes.^65^

Another well‐known carrier, breast cancer resistant protein coded by ABCG2 is found to be overexpressed in ovarian 66 and breast cancers.^67^ Upregulated BRCP is known to protect cancer cells from topotecan 66, 68 and mitoxantrone.^67^, ^68^ The mechanism of normal epithelial cells changes to drug resistant cancerous cells is by activating epithelial‐mesenchymal transition (EMT).^34^ Beginning of their transformation malignant epithelial cells go through angiogenesis and massive propagation.^69^ Cellular level changes occurring during the transition process transform epithelial cells to mesenchymal cells. These mesenchymal cells have anti apoptotic with increased migratory capacity and invasiveness.^70^ E‐cadherin a suppressor of insensitivity and motility is downregulated by transcription factors like Twist, Snail and Slug which are key coordinators for EMT.^71^ Snail and Twist are overexpressed in paclitaxel‐resistant EOC cells which is predicted by the molecular level modifications during EMT.^72^, ^73^

Since the EMT is mediated by several signaling pathways,^74^ it has become clearer that by halting these pathways, EMT can be reverted as well as some biological effects like drug sensitivity.^75^ Overexpression of endothelin‐1 and endothelin A receptor has been shown to enable EOC cells with increased resistance to chemo‐drugs, and thus, increase their relative survival capacity.^76^ In advanced‐stage EOC, ET (Endothelin) 1 and ET A receptor, ETAR pathways are overexpressed.^77^ The ET‐1 and ETAR are overexpressed with increased MAP kinase (MAPK) and protein kinase B phosphorylation, c‐1ell proliferation, in drug resistant EOC cells.^76^ In a study, treatment of cancer cells with the drugs that can block ETAR‐driven EMT, inhibition of tumor progression was seen, and chemo resistance has been overcome. EMT markers are used as a tool in several randomized clinical trials to develop personalized therapies. Clinical trial based on the aspirin treatment (NCT02602938) for metastatic breast and colorectal is an example of circulating tumor cells (CTC)s with EMT features.^78^

## 8 IMMUNOTHERAPIES

Immunotherapy involves various methods enhancing immune system. Exploiting the immune system for tumor recession is an ancient procedure as in 2600 BC The Pharaoh Imhotep self‐infected to enable tumor recession.^79^ Native and adaptive immunity of the patient are activated in the beginning of the tumor formation.^80^, ^81^ In the later stages of tumor growth, the tumor microenvironment inhibits the immune system in targeting cancer.

Anti‐tumor lymphocytes from healthy adults and patients are used in treatment using adoptive cell transfer to stimulate cancer decline.^82^ In this approach, the autologous T cells are collected from patient's peripheral blood or resected tumor tissue or tumor‐infiltrating lymphocytes (TIL) and those cells are expanded or manipulated ex vivo, an environment different from the patients tumor microenvironment (TME). These T cells cultured ex vivo and recombinant interleukin 2 are given back to patients.^83^

Use of cancer vaccines is another approach to bring about immune activation. T‐cell responses were stimulated by activating the antigen‐presenting cells.^84^ More recently, 11 heavily treated patients with platinum‐resistant OC (PROC) have been treated with GL‐ONC1 on a phase Ib protocol [NCT02759588]. GL‐ONC1 is a modified vaccinia virus developed by Genelux Corporation (San Diego, CA) that causes tumor cell oncolysis, immune activation through release of oncoproteins, presentation of both foreign and tumor antigens by dendritic cells, and durable anti‐cancer T cell tumor‐specific memory. In this phase Ib trial [NCT02759588], patients received a minimum of three prior lines of therapy, with five patients having had at least five prior lines. Nine patients had PROC, one was platinum refractory, and one was intermediate platinum sensitive (7 months prior PFS). Ten patients progressed on their prior line of therapy and nine had ascites or pleural effusions. The trial involved intraperitoneal (IP) infusion of GL‐ONC1 monotherapy that was given at higher dosages and contained provisions for dose‐escalation every three patients. Two separate IP instillations were performed 24 hours apart through a tunneled catheter system. The primary objectives were measurement of toxicity and secondary endpoints were anti‐tumor response. Encouraged by these preliminary outcomes, a clinical trial for first dose cohort is currently underway.

Manipulation of immune checkpoints has become the modern revolution in cancer immunotherapy. Cytotoxic T‐Lymphocyte‐Associated Protein 4 (CTLA‐4) and Programmed Cell Death Protein 1 (PD‐1) are Immune checkpoint linked to T cells. These proteins control the equilibrium of immune response and tolerance upon influencing the T lymphocyte activity. Activated T lymphocyte functions as an inhibitor via “negative feedback loop” mechanism and protect normal tissues from tumor‐derived immune response.^85^ These proteins are over expressed in OC patients and their natural anti‐cancer immunity is at disadvantage. Currently, several monoclonal antibodies (mAb) against CTLA‐4 and other proteins and its ligand are used in clinics.^86^, ^87^, ^88^ FDA approved mAb's targeted against immune checkpoints for various cancers are given in Table 1.

**Table 1:** Currently approved monoclonal antibodies targeted against Immune checkpoint proteins

Drug name	Immune checkpoint target	Current approval as of June, 2019
Ipilimumab	CTLA‐4	Melanoma, renal cell carcinoma (combined with nivolumab), colorectal cancer
Pembrolizumab	PD‐1	Nonsmall cell lung cancer (NSCLC), squamous cell carcinoma of head and neck (SCCHN), classic Hodgkin's lymphoma, large B‐cell lymphoma, urothelial cancer, micro‐satellite instability‐ high (MSI_H)or mismatch repair deficient (dMMR) cancers, gastric or GEJ adenocarcinoma and cervical ca
Nivolumab	PD‐1	NSCLC, melanoma, RCC, classic HL, squamous cell carcinoma of head and neck (SCCHN), urothelial cancer (UC), MSI‐H or dMMR colorectal cancer, hepatocellular cancer
Avelumab	PD‐L1	Merkel cell carcinoma (MCC), UC
Durvalumab	PD‐L1	UC, NSCLC
Atezolizumab	PD‐L1	UC, NSCLC
Tremelimumab	CTLA‐4	Awaiting approval

Abbreviations: CTLA‐4, cytotoxic T lymphocyte‐associated antigen 4; PD‐1: programmed cell death 1; PD‐L1: programmed cell death ligand 1.

## 9 IMMUNOTHERAPY FOR OC

**Figure 3:**
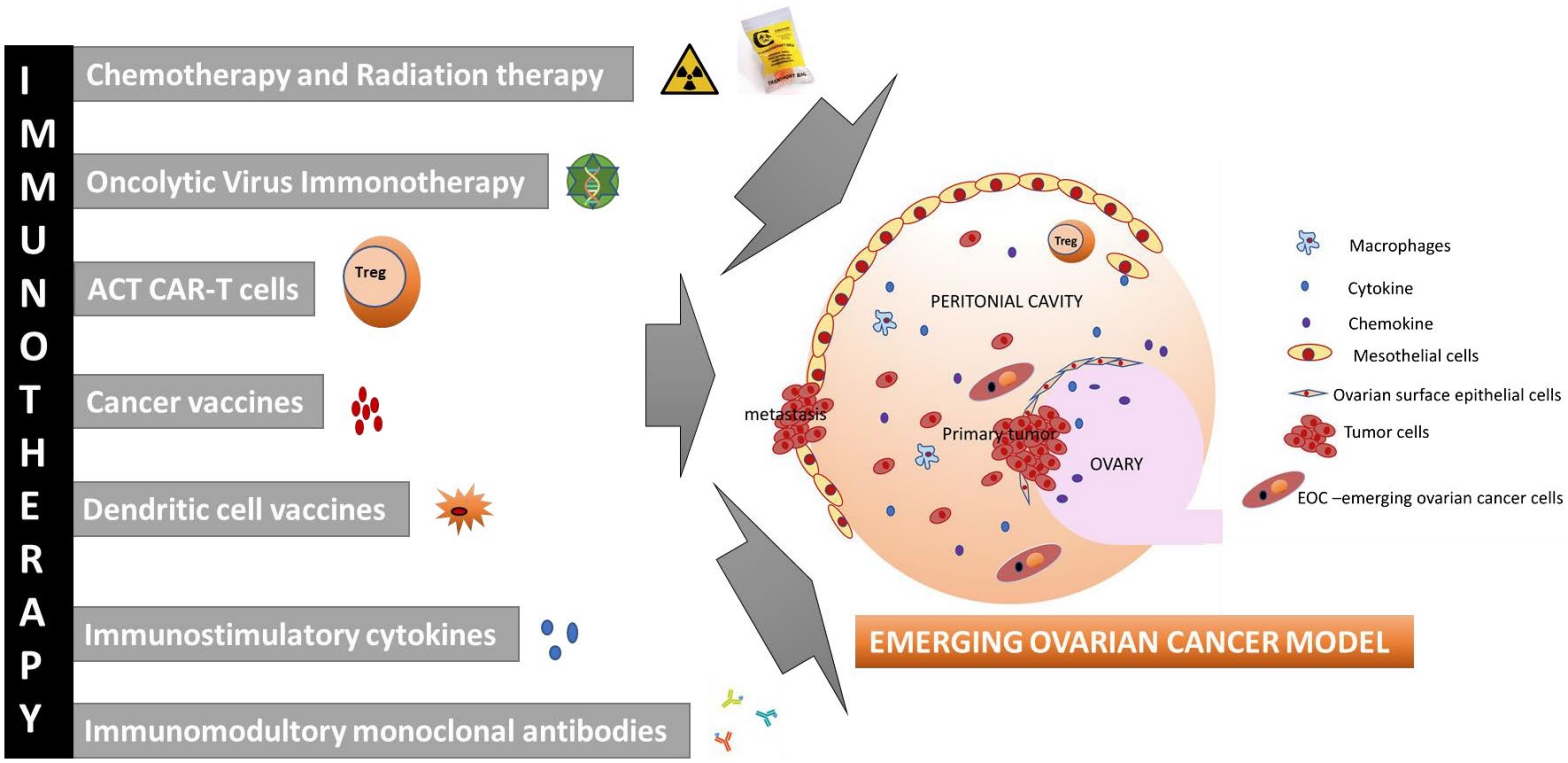
Emerging immunotherapies for ovarian cancer. A schematic representation with the details on cancer vaccines, dendritic cell vaccines, adoptive T‐cell transfer, immunostimulatory cytokines are some of the techniques explained in this review

The success rate for immunotherapy for OC treatment is very low and there is not yet any FDA approval for immune therapies for OC. Motivated by some of the recent encouraging results in other closely‐related tumor types, the scientific community has also started adapting immunotherapy to treat gynecologic cancers. Antibodies and T cells responsive for cancer are detected from ascites, blood, and tumor of advanced‐stage OC patients.^89^ Since it is known that the TILs expression level is linked to increased survival rate in OC patients, immunotherapies are highly potential for effective treatment outcomes, similar to other cancers.^90^ Table S1 includes current clinical trials listed on http://www.ClinicalTrial.gov that are recruiting patients. Schematic representation for the emerging immunotherapies for OC is given in Figure 3.

## 10 INHIBITORS AND MODULATOR

Important factors that help predict the tumor responses to inhibitors for immune checkpoint are the effector immune cell availability, approachability, and the tumor dependence on immune checkpoint pathways. TILs and PD‐L1 are identified as the markers to predict immune response to inhibitors. Using these markers, higher than 50% of advanced SOC estimated to act in response to immune checkpoint inhibitors via adaptive immune resistance. It is nearly absent low‐grade SOC, about 25% in other pathological types cancers.^91^ Currently, several immune checkpoint inhibitors are tested in animal studies and some of them are in clinical studies for OC treatment.^82^, ^92^

Preliminary data from clinical studies with all the current inhibitors reveals limited efficacy in OC with 15% rates of objective response, ORR. Hence it is very important future studies are required to identify more biomarkers for immune checkpoint inhibitors. 17% of ORR with a rate of 83% disease control is reported for a phase I study of olaparib (Poly ADP ribose polymerase [PARP] inhibitor) and durvalumab (anti‐PD‐L1).^93^ Table S3 shows the currently recruiting clinical trials (as of August, 2019) that utilizes immunotherapy for patients with OC.

## 11 VACCINES USED IN OC THERAPY

Vaccines used in cancer therapy will activate the immune cells for the elimination of cancerous cells. Specific tumor‐associate antigens (TAAs) are administered using various methods. Some of the common vaccines tested as cancer vaccines are developed using various methods, epigenetic, and genetic. Vaccines are administered as it or along with cytokines or other accelerating factors.^94^

CA125, p53 protein, folate receptor‐alpha (FRα), human epidermal growth factor receptor‐2 (HER2), and cancer‐testis antigens, like melanoma‐associated antigen A4 (MAGE‐A4) and New York‐esophageal squamous cell carcinoma11 (NY‐ESO‐1) are potential TAA molecules found in OC..^95^ Cancer Vaccine therapeutic investigation is an actively growing area in OC research. Currently, there are mainly pilot and phase I or II trials on the use of therapeutic vaccines in OC.^85^, ^96^ A list of ongoing vaccine studies in OC (Source: http://www.ClinicalTrial.gov) is given in Table S4.

NY‐ESO‐1, a potential molecule for targeted vaccine due to its over expression in OC exhibited stimulated immune response specific to T cells.^97^ Combination therapy of NY‐ESO‐1 with DNA methylation inhibitors and chemotherapy was administered in patients with recurrent disease for therapeutic efficacy enhancement.^83^ This increases the NY‐ESO‐1 antibody availability, T‐cell responses that lead to clinical response in OC patients.

As noted earlier, oncolytic virus (OV) has shown synergy when combined with checkpoint inhibitor antibodies.^98^ Oncolytic virus immunotherapy or combination of OV with other molecules that are immune‐stimulatory or induce immunogenic responses are encouraging avenues to explore as novel therapeutic options for OC.^82^

Her2/Neu, tumor antigen presents in almost 90% of the recurrent OC cases. Clinical study of 11 patients were given Her2/Neu packed antigen autologous dendritic cells combined with antigens of telomerase reverse transcriptase (human) and pan‐DR peptides.^99^ 90% 3‐year overall survival was reported as the outcome for this study of patients in remission with advanced OC.

Whole tumor cell vaccines will induce immunologic reaction for a larger range of antigens compared to specific TAA.^100^ Broader reaction with T cells can also be induced using whole vaccine.^100^ Another important avenue in vaccine therapy is personalized peptide vaccines developed from individual tumor depending on the human leukocyte antigen and IgG expressions.^101^ Clinical research showed that patient with Pt‐ sensitive cancer have 39.3 months overall survival rate. For patients with Pt resistant cancer overall survival rate is 16.2 months.

Another novel therapeutic method is to explore the use cancer cells from the patient to deliver viruses to the tumor.^102^ Tumor microenvironment can be manipulated using cancer cells as virus vehicles. Changing a “cold” cancer to a “hot” cancer potentiates anti‐cancer reaction. This a promising strategy for OC patients who do not get benefitted from current therapies due to suboptimal immune infiltration. Schematic representation for the emerging immunotherapies for OC is given in Figure 3.

## 12 ADOPTIVE CELLULAR THERAPY‐ADOPTIVE T‐CELL TRANSFER

Peripheral blood lymphocytes (PBLs) are separated from patients’ blood and will be used for the isolation of tumor‐specific lymphocytes. Tumor‐specific PBLs will be grown to supply back to the patient. Anti‐cancer action of PBLs can also be enhanced by genetic modification.^103^ Clinical trials of ACT for OC are ongoing. ACT has shown around 72% reaction rates that last for more than three years in metastatic melanoma occurring at.^83^ This is a very promising outcome that can be translated into OC therapy by optimizing the conditions for Adoptive cellular therapy (ACT) in OC therapy.

## 13 CAR‐T‐CHIMERIC ANTIGEN RECEPTOR T

The major limitation to the ACT trials in the beginning stages were the need for isolation and culturing of functional cancer responsive T cells. The emergence of engineered T cells has become a promising tool to enhance the cancer immune therapy.^82^, ^104^, ^105^ Tumor‐specific targeting can be gained using patients receptors of T cell and CARs. The tumor recognition in a major histocompatibility complex (MHC) can be achieved by CAR‐T cells. T‐cell stimulation and selectivity of antigen features are combined in one combination molecule.^105^ The initial group of CARs was examined in OC and other tumors inducing modest responses.^105^, ^106^ At the very first CAR T‐cells trial in OC, cancer load was not reduced in patients.^106^ FRα,^107^ HER‐2,^108^ CA125 (MUC16),^109^, ^110^ and mesothelin 111 are ensuring antigens for CARS. CAR‐T therapeutic efficacy still need to be improved in OC. Combination treatment modalities to overcome these problems may be a novel approach. Combining inhibitors for immune checkpoint with CAR‐T cells is a better therapeutic option for OC.^112^ A schematic representation of emerging immunotherapies for OC is given in Figure 3.

## 14 FUTURE PERSPECTIVES

Regardless of the extensive developments in OC therapy, it is still the deadliest malignancy in women. The biggest hurdle is the shortage of efficient screening procedure that helps to detect the tumor at an early stage. Even though the curing rate for beginning stage OC patient is 90%, about 20% of OC is detected as early as stage1. This reveals the need for future research for finding biomarkers that are more responsive and specific for detection of OC at an early stage.

Surgery and chemotherapy are conventional therapy for ovarian carcinoma. Poor prognosis with a recurring progressive cancer is the major challenge to the treatment. The prognosis varies for each patient and it depends on the level of response to preliminary therapy. Drug administration by IP is the efficient way to target OC cells situated the peritoneal area. Removal of all the residual tissues by surgery followed by chemotherapy, is the most ideal cure for OC.

Sensitivity to chemotherapeutic agents is a crucial parameter in therapeutic efficacy. Research to identify biomarkers for apoptosis and chemo resistance in OC therapy should be one of the prime goals in cancer research (eg, Caris^®^ Assays). Newly identified microRNA biomarkers linked to platinum drug resistance are let‐7^109^ and ATP11B.^108^ Another upcoming are of research in OC is combination therapy of drugs and other small molecules that can enhance therapeutic efficacy by increasing drug sensitivity and reducing drug resistance. Currently, several studies are in progress with the compounds that modify Bcl2 proteins family,^82^, ^85^ and agents for targeting DNA repair and inhibit PARP.^113^, ^114^, ^115^ To improve drug sensitivity to cisplatin, small molecules like triethylenetetramine, genistein, butathione sulfoximine, and rapamycin are under the stage of preclinical testing.^116^ Triethylenetetramine inhibits telomerase, induces anti‐antiogenesis and acts as anti‐cancer agent. Triethylenetetramine reversed cisplatin resistance in OC cells. Genistein showed anti‐cancer activity in both pediatric and adult cancer models. Triethylenetetramine sensitized OC cells against cisplatin. Butathione sulfoximine also showed sensitizing effects in gastric and OC cells against cisplatin. Laboratory studies also demonstrated the effect of rapamycin for inducing the effect of cisplatin in OC breast cancer and lung cancer cells.

Another promising technique for OC therapy is silencing gene expression. By this technique, specific sets of genes can be targeted and altered, and it requires less dosage. This technique is still the subject of ongoing research to overcome its challenges like stability and compound delivery to a target site.^110^, ^111^ MicroRNAs are used as targeted molecules for diagnosis is another growing field. Developing clinical trials with more molecules similar to what we explained above will help to overcome the challenges in OC therapy.

Diab et al have done a review of targeted therapy for OC for 2010‐2017.^117^ Targeted therapies are evolving in three main fields, angiogenesis, signaling, and apoptosis. The VEGF pathway is focused for angiogenesis. PI3K/Akt and the MAP kinase pathways critically involved signaling cascades. McCabe et al^118^ have conducted examination of trials in EOC to investigate the association between platinum‐resistance and response to anti‐angiogenic agents. The analysis revealed that novel anti‐angiogenic therapies would be beneficiary for the patients with platinum‐resistant EOC.

**Figure 4:**
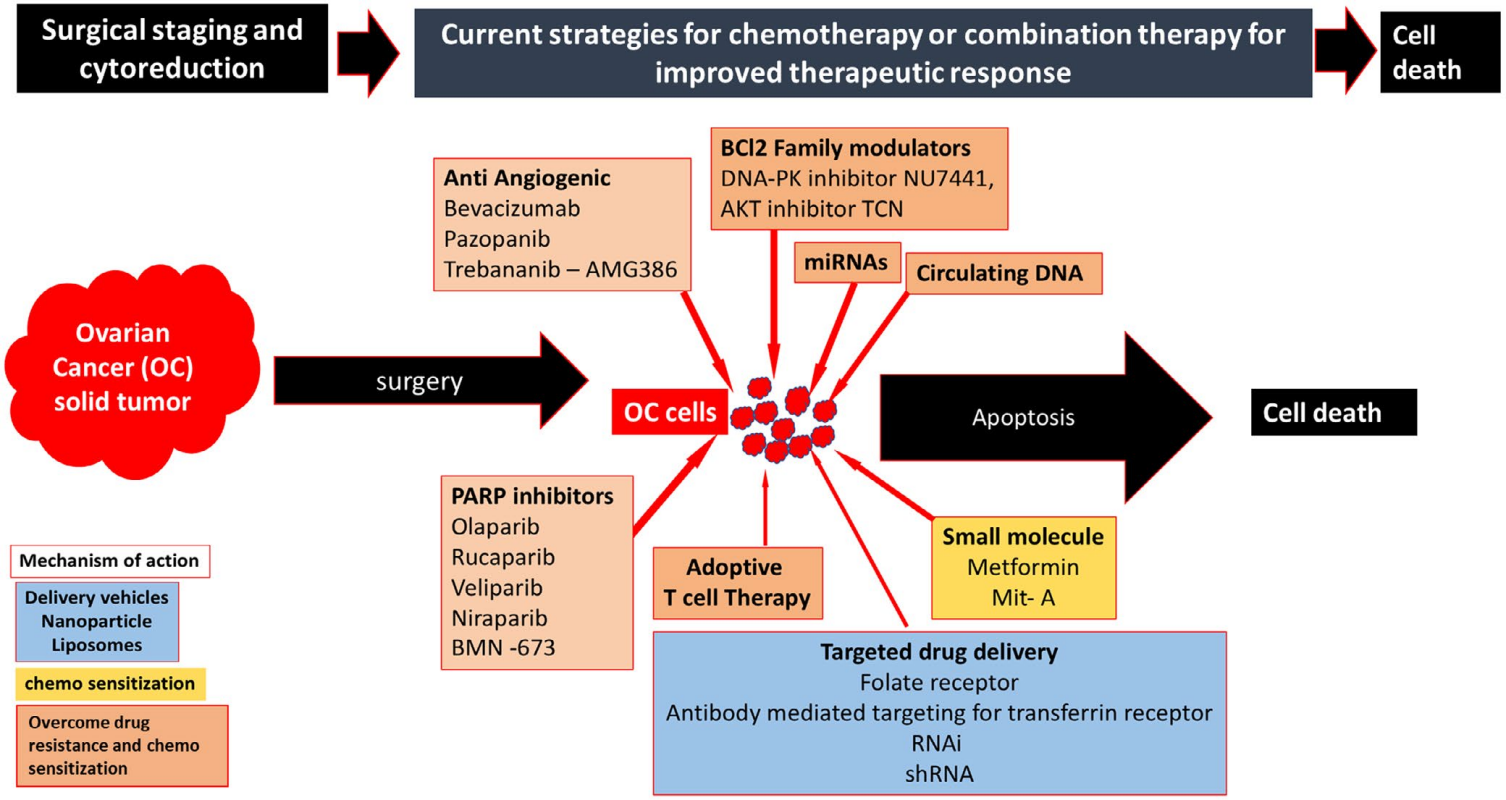
Current strategies for improving therapeutic response. Effective Targeted therapy, Usage of PARP inhibitors, combination therapy, immunotherapy, and usage of chemosensitizers are some of the future strategies for improved therapeutic responses for ovarian cancer treatment

Dose‐dense chemotherapy is the promising option currently available for the patients with poor responses to chemotherapy. PARP inhibitors are the most emerging class of new drugs in combination therapy with the traditional chemotherapy drugs listed in Figure 3. Bevacizumab is recently approved for EOC treatment. The common challenge with anti‐angiogenic agents is the non‐availability of efficient biomarkers. Folate receptor targeting required further research to consider as one of the treatment options. Despite cost issues, regular Breast cancer susceptibility gene (BRCA) screening should be done for all OC patients for a better selection of targeted therapy. Knowledge about tumor microenvironment and immune suppressive pathways are crucial for newer immunotherapeutic approaches toward OC. For a personalized medical treatment, systematic data analysis of molecular and genetical categorization of various types of OC with precision is required.^119^ A schematic representation of some of the most common strategies for improving therapeutic responses is given in Figure 4.

## 15 CONCLUSIONS

Ovarian cancer affects the lives of many women around us. Despite continued efforts and steady improvements in treatment over the past few decades, OC still remains the deadliest malignancy in women. The poor clinical outcome is due to the deficiency of effective tools for detecting the disease at an early stage, chemotherapy resistance and increased heterogeneity of the disease. The vast majority of cases have high‐grade papillary serous histology marked by p53 mutations and 25% of cases have either inherited or acquired mutations in BRCA. Primary therapy is initiated with cytoreductive surgery and chemotherapy. Even with optimization of treatment protocols that have improved PFS, only limited gains in OS. Ultimately, approximately 80% of patients develop PROC. Once this occurs, further chemotherapy response rates are about 10%‐15% and survival averages 9‐12 months. Therefore, we are critically in need of developing novel therapies to improve cure rates and provide effective long‐term disease stability for PROC.

Targeted therapy is the fast growing modalities for cancer treatment. For targeted therapy drugs or small molecules will be used to block tumor growth. More studies should be done on combination therapies involving one or more of these small molecules as modulators for OC treatment. Such research should be augmented to fight chemo resistance better treatment outcomes. Inhibitors for various genes involved in the signal pathway in tumor growth should be another area of focus for future studies for OC treatment. Study of the various molecules involved in tumor micro environment at various stages of tumor metastasis is crucial for the development of better immunotherapies for OC.^120^ How each one of these molecules control various treatment strategies and the immune system of the patient. Immune and cellular therapies coupled with genetic testing and precision assays (biomarkers) are promising strategies for better clinical outcomes. Novel strategies and rapid growth of research in medical field will lead to better therapeutic schemes to minimize ill health and improved life expectancy for patients with OC.

## CONFLICT OF INTEREST

None declared.
