# Supplementary material for: Ovarian cancer: Current status and strategies for improving therapeutic outcomes
Source: Cancer Med. 2019 Sep 27;8(16):7018–31. doi: 10.1002/cam4.2560 (PMC6853829; doi:10.1002/cam4.2560)
Supplement: Supplementary file 1 [file CAM4-8-7018-s001.docx]

**Table S1. A summary of ongoing biomarker studies in Ovarian Cancer (Source: Clinical trial.gov)**

|  | **NCT Number** | **Title** | **Status** | **Indications** | **Interventions** |
| --- | --- | --- | --- | --- | --- |
|  |  |  |  |  |  |
| 1 | NCT03010124 | [Prognostic and Predictive Biomarkers in Ovarian Cancers](https://clinicaltrials.gov/ct2/show/NCT03010124?term=Biomarkers&recrs=ab&cond=ovarian+cancer&draw=2&rank=4) | Recruiting | Ovarian Cancer | Procedure: Blood sample |
|  |  |  |  |  |  |
| 4 | NCT03112733 | [Serum Biomarkers in Diagnosis and Predicting Prognosis of Ovarian Cancers](https://clinicaltrials.gov/ct2/show/NCT03112733?term=Biomarkers&recrs=ab&cond=ovarian+cancer&draw=2&rank=5) | Recruiting | Endometrial Neoplasms  Ovarian Neoplasms | Diagnostic Test: TFF3, SFRP4, Romo1, NFKB |
| 3 | NCT03004391 | [Development of a Decision Aid to Facilitate Ovarian Cancer Patient's Choices Regarding Biomarker CA125](https://clinicaltrials.gov/ct2/show/NCT03004391?term=Biomarkers&recrs=ab&cond=ovarian+cancer&draw=2&rank=6) | Recruiting | Ovarian Cancer |  |
|  |  |  |  |  |  |
| 4 | NCT03150121 | [Biomarkers for Early Detection of Ovarian Cancer Using Uterine Lavage](https://clinicaltrials.gov/ct2/show/NCT03150121?term=Biomarkers&recrs=ab&cond=ovarian+cancer&draw=2&rank=7) | Recruiting | Ovarian Cancer  Ovarian Carcinoma  Fallopian Tube Cancer | Procedure: Uterine lavage  Procedure: Blood sample  Device: Uterine lavage catheter |
| 5 | NCT03419689 | [Study Looking at Biomarkers in Ovarian Cancer](https://clinicaltrials.gov/ct2/show/NCT03419689?term=Biomarkers&recrs=ab&cond=ovarian+cancer&draw=2&rank=8) | Recruiting | Gynecologic Cancer | Procedure: Tumor tissue collection  Procedure: Blood sample collection  Procedure: Ascites Collection  Procedure: Fluid Collection |
| 6 | NCT01000259 | [Study of Tumor Tissue Samples From Patients Who Have Undergone Surgery for Advanced Stage III or Stage IV Ovarian Epithelial Cancer](https://clinicaltrials.gov/ct2/show/NCT01000259?term=Biomarkers&recrs=ab&cond=ovarian+cancer&draw=2&rank=9) | Recruiting | Stage IIIA Ovarian Cancer  Stage IIIB Ovarian Cancer  Stage IIIC Ovarian Cancer  Stage IV Ovarian Cancer | Other: Laboratory **Biomarker** Analysis |
| 7 | NCT02520115 | [Folate Receptor in Diagnosing Ovarian Cancer Using Serum Samples From Patients With Newly Diagnosed Pelvic Mass or Previously Diagnosed Ovarian Cancer](https://clinicaltrials.gov/ct2/show/NCT02520115?term=Biomarkers&recrs=ab&cond=ovarian+cancer&draw=2&rank=12) | Recruiting | Anastomotic Leak  Adnexal Mass  Borderline Ovarian Epithelial Tumor  Ovarian Clear Cell Tumor  (and 4 more...) | Drug: Dexamethasone  Other: Laboratory **Biomarker** Analysis  Drug: Valproic Acid |
| 8 | NCT03378297 | [IMPACT: A Non-randomized WOO Study of Novel Therapeutic Agents in Women Triaged to Primary Surgery for EOC](https://clinicaltrials.gov/ct2/show/NCT03378297?term=Biomarkers&recrs=ab&cond=ovarian+cancer&draw=2&rank=14) | Recruiting | Ovarian Cancer | Drug: Metformin  Drug: Acetylsalicylic acid  Drug: Olaparib  Drug: Letrozole |
| 9 | NCT02296307 | [DOvEE - Diagnosing Ovarian & Endometrial Cancer Early](https://clinicaltrials.gov/ct2/show/NCT02296307?term=Biomarkers&recrs=ab&cond=ovarian+cancer&draw=2&rank=15) | Recruiting | Ovarian Neoplasms | Other: Blood test: CA-125 **biomarker**  Other: Second Test: Transvaginal Ultrasound  Other: Follow-up phone call |
| 10 | NCT02687321 | [The Role of HE4 in the Follow-up of Advanced Ovarian, Fallopian Tube and Primary Peritoneal Cancer](https://clinicaltrials.gov/ct2/show/NCT02687321?term=Biomarkers&recrs=ab&cond=ovarian+cancer&draw=2&rank=16) | Recruiting | Ovarian Neoplasms | Other: Determination of CA 125 and HE4, Computed tomography |
| 11 | NCT02595281 | Determination of the Interest of HE4 as a Relapse Biomarker in Ovarian Cancers Stages IIIb, IIIc and IV After Neo-adjuvant Chemotherapy and Surgery. | Recruiting | Ovarian Cancer | HE 4 |
| 12 | NCT03556566 | [Open Label Immunotherapy Trial for Ovarian Cancer](https://clinicaltrials.gov/ct2/show/NCT03556566?term=Biomarkers&recrs=ab&cond=ovarian+cancer&draw=2&rank=17) | Recruiting | Ovarian Cancer | Biological: Tableted vaccine (V3-OVA) containing ovarian cancer antigens |

| 13 | NCT00005095 | [Specimen and Data Study for Ovarian Cancer Early Detection and Prevention](https://clinicaltrials.gov/ct2/show/NCT00005095?term=Biomarkers&recrs=ab&cond=ovarian+cancer&draw=2&rank=18) | Recruiting | Cervical Cancer  Endometrial Cancer  Fallopian Tube Cancer  (and 6 more...) | Other: laboratory **biomarker** analysis  Other: screening questionnaire administration  Procedure: study of high risk factors |
| --- | --- | --- | --- | --- | --- |
|  |  |  |  |  |  |
| 14 | NCT02302742 | [Triple Negative Breast Cancer and Germline Hereditary Breast and Ovarian Cancer Mutation Carrier Registry](https://clinicaltrials.gov/ct2/show/NCT02302742?term=Biomarkers&recrs=ab&cond=ovarian+cancer&draw=2&rank=19) | Recruiting | Breast Cancer  Hereditary Breast and Ovarian Cancer |  |
|  |  |  |  |  |  |
| 15 | NCT03456700 | [Auranofin and Sirolimus in Treating Participants With Ovarian Cancer](https://clinicaltrials.gov/ct2/show/NCT03456700?term=Biomarkers&recrs=ab&cond=ovarian+cancer&draw=2&rank=20) | Recruiting | Ovarian Serous Tumor  Recurrent Ovarian Carcinoma | Drug: Auranofin  Other: Laboratory Biomarker Analysis |
|  |  |  |  |  | Drug: Sirolimus |
| 16 |  | [Phase Ib Study of Chiauranib in Patients With Ovarian Cancer](https://clinicaltrials.gov/ct2/show/NCT03166891?term=Biomarkers&recrs=ab&cond=ovarian+cancer&draw=2&rank=21) | Recruiting | Ovarian Cancer | Drug: Chiauranib |
|  |  |  |  |  |  |
| 17 | NCT03166891 | [p53 Activation in Platinum-Resistant High Grade Serous Ovarian Cancer, a Study of PLD With APR-246](https://clinicaltrials.gov/ct2/show/NCT03268382?term=Biomarkers&recrs=ab&cond=ovarian+cancer&draw=2&rank=22) | Recruiting | High-grade Serous Ovarian Cancer | Drug: APR-246  Drug: Pegylated Liposomal Doxorubicin Hydrochloride (PLD) |
|  |  |  |  |  |  |
| 18 | NCT01889615 | [Dual PETOvac - Dual Time PET/CT in the Preoperative Assessment of Ovarian Cancer](https://clinicaltrials.gov/ct2/show/NCT01889615?term=Biomarkers&recrs=ab&cond=ovarian+cancer&draw=2&rank=23) | Recruiting | Ovarian Cancer | Other: PET/CT(3hours] |
|  |  |  |  |  |  |
| 19 | NCT02109367 | [Diagnostic Utility of Culdocentesis in Patients With a Suspicious Adnexal Mass](https://clinicaltrials.gov/ct2/show/NCT02109367?term=Biomarkers&recrs=ab&cond=ovarian+cancer&draw=2&rank=24) | Recruiting | Ovarian Cancer |  |
|  |  |  |  |  |  |
| 20 | NCT03297489 | [Intravital Microscopy in Evaluating Patients With Primary Peritoneal, Fallopian Tube, or Stage IA-IV Ovarian Cancer](https://clinicaltrials.gov/ct2/show/NCT03297489?term=Biomarkers&recrs=ab&cond=ovarian+cancer&draw=2&rank=25) | Recruiting | Fallopian Tube Carcinoma | Procedure: Diagnostic Microscopy |
|  |  |  |  | Primary Peritoneal Carcinoma  Stage I Ovarian Cancer  (and 12 more...) | Drug: Fluorescein Sodium Injection  Other: Laboratory Biomarker Analysis |
|  |  |  |  |  |  |

"
