# Supplementary material for: Ovarian cancer: Current status and strategies for improving therapeutic outcomes
Source: Cancer Med. 2019 Sep 27;8(16):7018–31. doi: 10.1002/cam4.2560 (PMC6853829; doi:10.1002/cam4.2560)
Supplement: Supplementary file 2 [file CAM4-8-7018-s002.docx]

Table S2. A list of currently approved chemotherapeutic agents for ovarian cancer chemotherapy and their mechanism of anticancer activity

| Drug | Mechanism |
| --- | --- |
| Alkeran (Melphalan) | DNA alkylation |
| Avastin (Bevacizumab) | Anti angiogenic agent |
| Bevacizumab |  |
| Carboplatin | DNA alkylation |
| Cisplatin |  |
| Cyclophosphamide | DNA alkylation |
| Doxorubicin Hydrochloride | forms complexes with DNA by intercalation |
| Dox-SL (Doxorubicin Hydrochloride Liposome) | forms complexes with DNA by intercalation |
| DOXIL (Doxorubicin Hydrochloride Liposome) |  |
| Doxorubicin Hydrochloride Liposome |  |
| Evacet (Doxorubicin Hydrochloride Liposome) |  |
| LipoDox (Doxorubicin Hydrochloride Liposome) |  |
| Gemcitabine Hydrochloride | Inhibits thymidylate synthetase, leading to inhibition of DNA synthesis and cell death |
| Gemzar (Gemcitabine Hydrochloride) |  |
| Hycamtin (Topotecan Hydrochloride) | Topoisomerase Inhibitor (DNA) |
| Lynparza (Olaparib) | PARPi |
| Niraparib Tosylate Monohydrate | PARPi |
| Zejula (Niraparib Tosylate Monohydrate) |  |
| Rubraca (Rucaparib Camsylate) | PARPi |
| Rucaparib Camsylate |  |
| Paclitaxel | Induced mitotic arrest |
| Taxol |  |
| Thiotepa | DNA alkylation |
| Bleomycin sulfate | Bleomycin sulfate forms complexes with iron that reduce molecular oxygen to superoxide and hydroxyl radicals which cause single- and double-stranded breaks in DNA; these reactive oxygen species also induce lipid peroxidation, carbohydrate oxidation, and alterations in prostaglandin synthesis and degradation. |
| Etoposide Phosphate | Etoposide binds to the enzyme topoisomerase II, inducing double-strand DNA breaks, inhibiting DNA repair, and resulting in decreased DNA synthesis and tumor cell proliferation. |
| Vinblastine | Vinblastine disrupts microtubule formation |

"
