# Supplementary material for: Ovarian cancer: Current status and strategies for improving therapeutic outcomes
Source: Cancer Med. 2019 Sep 27;8(16):7018–31. doi: 10.1002/cam4.2560 (PMC6853829; doi:10.1002/cam4.2560)
Supplement: Supplementary file 3 [file CAM4-8-7018-s003.docx]

**Table S3. Immunotherapy Studies in Ovarian Cancer**

*Information Sources: Clinical trial.gov; U.S. National Library of Medicine; U.S. National Institutes of Health | U.S. Department of Health & Human Services*

|  | **NCT Number** | **Title** | **Status** | **Study Results** | **Indications** | **Interventions** |
| --- | --- | --- | --- | --- | --- | --- |
| 1 | NCT03989336 | A Phase I/II, Open-label, Two-arm, Single-center Study to Evaluate the Safety and Efficacy of the Pan-immunotherapy in Subjects With Relapsed/Refractory Ovarian Cancer | Recruiting | No Results Available | - Ovarian Cancer | - Parallel Assignment |
| 2 | NCT02849353 | Combination of Cryosurgey and NK Immunotherapy for Recurrent Ovarian Cancer | Recruiting | No Results Available | • Recurrent Ovarian Cancer | • Device: Cryosurgery • Biological: NK immunotherapy |
| 3 | NCT03556566 | Open Label Immunotherapy Trial for Ovarian Cancer | Recruiting | No Results Available | • Ovarian Cancer | • Biological: Tableted vaccine (V3-OVA) containing ovarian cancer antigens |
| 4 | NCT02759588 | GL-ONC1 Oncolytic Immunotherapy in Patients With Recurrent Ovarian Cancer | Recruiting | No Results Available | • Ovarian Cancer • Peritoneal Carcinomatosis • Fallopian Tube Cancer | • Biological: GL-ONC1 |
| 5 | NCT02132988 | Trial of Active Immunotherapy With Globo H-KLH (OPT-822/821) in Women Who Have Non-Progressive Ovarian Cancer | Recruiting | No Results Available | • Ovarian Cancer | • Biological: OPT-822/OPT-821 |
| 6 | NCT03318900 | T Cell Immunotherapy for Advanced Ovarian Cancer | Recruiting | No Results Available | • Platinum Resistant Ovarian Cancer | • Procedure: Leukapheresis • Drug: Utomilumab • Drug: Cyclophosphamide • Biological: T Cell Infusion • Drug: Interleukin-2 |
| 7 | NCT03249142 | Immunotherapy With Neo-adjuvant Chemotherapy for Ovarian Cancer | Recruiting | No Results Available | • Ovarian Cancer | • Drug: ARM A Durvalumab/chemotherapy association • Drug: ARM B Durvalumab/Tremelimumab/ chemotherapy association |
| 8 | NCT03100006 | Phase Ib/IIa Trial to Evaluate Oregovomab and Nivolumab in Epithelial Cancer of Ovarian, Tubal or Peritoneal Origin | Recruiting | No Results Available | • Epithelial Ovarian Cancer | • Drug: Nivolumab • Drug: Oregovomab |
| 9 | NCT03162562 | The Safety and Antitumor Activity of the Combination of Oregovomab and Hiltonol in Recurrent Advanced Ovarian Cancer | Recruiting | No Results Available | • Cancer of Ovary • Neoplasms, Ovarian • Ovarian Cancer Stage IV • Ovarian Cancer Recurrent • Ovarian Cancer Stage III • Ovary Cancer | • Biological: Oregovomab • Drug: Poly ICLC |
| 10 | NCT02608684 | A Study of Pembrolizumab With Standard Treatment in Patients With Recurrent Platinum-resistant Ovarian Cancer | Recruiting | No Results Available | • Ovarian Cancer | • Drug: Pembrolizumab • Drug: Gemcitabine • Drug: Cisplatin |
| 11 | NCT03294694 | Ribociclib + PDR001 in Breast Cancer and Ovarian Cancer | Recruiting | No Results Available | • Metastatic Hormone-Receptor-Positive (HR+) Breast Cancer • HER2-Negative Breast Cancer • Metastatic Epithelial Ovarian Cancer | • Drug: Ribociclib • Drug: PDR001 • Drug: Fulvestrant |

| 12 | NCT03073525 | Trial of Atezolizumab and Vigil for Advanced Gynecological Cancers (A Companion Study to CL-PTL-119) | Recruiting | No Results Available | • Advanced Gynecological Cancers • Ovarian Cancer • Cervical Cancer • Uterine Cancer | • Biological: Vigil • Drug: Atezolizumab |
| --- | --- | --- | --- | --- | --- | --- |
| 13 | NCT03312114 | Anti—PD-L1 and SAbR for Ovarian Cancer | Recruiting | No Results Available | • Recurrent Epithelial Cancer of Ovary • Primary Peritoneal Carcinoma | • Drug: Avelumab |
| 14 | NCT02432378 | Intensive Locoregional Chemoimmunotherapy for Recurrent Ovarian Cancer Plus Intranodal DC Vaccines | Recruiting | No Results Available | • Cancer of Ovary • Cancer of the Ovary • Neoplasms, Ovarian • Ovarian Cancer • Ovary Cancer • Ovary Neoplasms | • Biological: Cisplatin + celecoxib + DC vaccine • Biological: Cisplatin + CKM + Celecoxib + DC Vaccine |
| 15 | NCT02948426 | Intraperitoneal Infusion of Autologous Monocytes With Sylatron (Peginterferon Alfa-2b) and Actimmune (Interferon Gamma-1b) in Women With Recurrent or Refractory Ovarian Cancer, Fallopian Tube Cancer or Primary Peritoneal Cancer | Recruiting | No Results Available | • Fallopian Tube Cancer • Ovarian Cancer • Primary Peritoneal Cancer | • Biological: Autologous Monocytes + ACTIMMUNE + SYLATRON |
| 16 | NCT03267589 | Trial in Patients With Relapsed Ovarian Cancer | Recruiting | No Results Available | • Ovarian Cancer | • Drug: Durvalumab, Tremelilumab, MEDI 9447, MEDI 0562 |
| 17 | NCT01583686 | CAR T Cell Receptor Immunotherapy Targeting Mesothelin for Patients With Metastatic Cancer | Recruiting | No Results Available | • Cervical Cancer • Pancreatic Cancer • Ovarian Cancer • Mesothelioma • Lung Cancer | • Drug: Fludarabine • Biological: Anti-mesothelin CAR transduced PBL • Drug: Cycolphosphamide • Drug: Aldesleukin |
| 18 | NCT03277482 | Durvalumab, Tremelimumab + Radiotherapy in Gynecologic Cancer | Recruiting | No Results Available | • Recurrent Gynecological Cancer • Metastatic Cervical Cancer • Metastatic Ovarian Cancer • Metastatic Vaginal Cancer • Metastatic Vulvar Cancer • Metastatic Endometrial Cancer • Recurrent Cervical Carcinoma • Recurrent Ovarian Carcinoma • Recurrent Vaginal Cancer • Recurrent Vulvar Cancer • Recurrent Endometrial Cancer | • Drug: Durvalumab • Drug: Tremelimumab • Radiation: Radiation Therapy |

| 19 | NCT03158935 | The ACTIVATE (Adoptive Cell Therapy InVigorated to Augment Tumor Eradication) Trial | Recruiting | No Results Available | • Advanced Ovarian Cancer • Malignant Melanoma | • Drug: Cyclophosphamide • Drug: Fludarabine • Procedure: Pembrolizumab • Biological: Tumor-Infiltrating Lymphocytes (TILs) • Biological: Interleukin-2 (IL-2) |
| --- | --- | --- | --- | --- | --- | --- |
| 20 | NCT02729298 | First-in-human Study of Oral TP-0903 (a Novel Inhibitor of AXL Kinase) in Patients With Advanced Solid Tumors | Recruiting | No Results Available | • Advanced Solid Tumors • EGFR Positive Non-small Cell Lung Cancer • Colorectal Carcinoma • Recurrent Ovarian Carcinoma • BRAF-Mutated Melanoma | • Drug: TP-0903 |
| 21 | NCT01376505 | Vaccine Therapy in Treating Patients With Metastatic Solid Tumors | Recruiting | No Results Available | • Malignant Solid Tumour • Breast Cancer • Malignant Tumor of Colon • GIST | • Biological: HER-2 vaccine • Biological: Extension HER-2 vaccine trial at OBD |
| 22 | NCT03018405 | A Dose Escalation Phase I Study to Assess the Safety and Clinical Activity of Multiple Cancer Indications | Recruiting | No Results Available | • Colorectal Cancer (CRC) • Ovarian Cancer (Epithelial and Fallopian Tube ) • Urothelial Carcinoma • Triple-negative Breast Cancer (TNBC) • Pancreatic Cancer • Acute Myeloid Leukemia/Myelodysplastic Syndrome • Multiple Myeloma (MM) | • Biological: NKR-2 cells |
| 23 | NCT02785250 | Study of DPX-Survivac Vaccine Therapy and Epacadostat in Patients With Recurrent Ovarian Cancer | Recruiting | No Results Available | • Recurrent Epithelial Ovarian Cancer • Recurrent Fallopian Tube Cancer • Recurrent Peritoneal Cancer | • Biological: DPX-Survivac • Drug: Cyclophosphamide • Drug: Epacadostat (INCB024360) |
| 24 | NCT02571725 | PARP-inhibition and CTLA-4 Blockade in BRCA-deficient Ovarian Cancer | Recruiting | No Results Available | • Ovarian Cancer • Fallopian Tube Cancer • Peritoneal Neoplasms | • Drug: Olaparib • Drug: Tremelimumab |
| 25 | NCT03300843 | Ability of a Dendritic Cell Vaccine to Immunize Melanoma or Epithelial Cancer Patients Against Defined Mutated Neoantigens Expressed by the Autologous Cancer | Recruiting | No Results Available | • Melanoma • Gastrointestinal Cancer • Breast Cancer • Ovarian Cancer • Pancreatic Cancer | • Biological: Peptide loaded dendritic cell vaccine |

| 26 | NCT03412877 | Administration of Autologous T-Cells Genetically Engineered to Express T-Cell Receptors Reactive Against Mutated Neoantigens in People With Metastatic Cancer | Recruiting | No Results Available | • Glioblastoma • Non-Small Cell Lung Cancer • Ovarian Cancer • Breast Cancer • Gastrointestinal/Genitourinary Cancer | • Drug: Cyclophosphamide • Drug: Fludarabine • Drug: Aldesleukin • Biological: Individual Patient TCR-Transduced PBL |
| --- | --- | --- | --- | --- | --- | --- |
| 27 | NCT02554812 | A Study Of Avelumab In Combination With Other Cancer Immunotherapies In Advanced Malignancies (JAVELIN Medley) | Recruiting | No Results Available | • Advanced Cancer | • Drug: Avelumab • Drug: Utomilumab • Drug: PF-04518600 • Drug: PD 0360324 |
| 28 | NCT02955251 | A Study of ABBV-428, an Immunotherapy, in Subjects With Advanced Solid Tumors | Recruiting | No Results Available | • Advanced Solid Tumors Cancer | • Drug: ABBV-428 • Drug: Nivolumab |
| 29 | NCT02457650 | T Cell Receptor-transduced T Cells Targeting NY-ESO-1 for Treatment of Patients With NY-ESO-1- Expressing Malignancies | Recruiting | No Results Available | • Bladder Carcinoma • Breast Cancer • Esophagus Carcinoma • Lung Cancer • Melanoma • Multiple Myeloma • Neuroblastoma • Ovarian Cancer • Synovial Sarcoma • Other Metastatic Solid Cancers | • Drug: Cyclophosphamide • Drug: Fludarabine • Biological: Anti-NY ESO-1 TCR-transduced T cells |
| 30 | NCT02766582 | Phase II: Pembrolizumab/Carboplatin/Taxol in Epithelial Ovary Cancer | Recruiting | No Results Available | • Ovarian Cancer | • Drug: Pembrolizumab • Drug: Carboplatin • Drug: Paclitaxel |
| 31 | NCT03250832 | Study of TSR-033 With an Anti-PD-1 | Recruiting | No Results Available | • Advanced Solid Tumors • Antibodies • Immunotherapy | • Drug: TSR-033 • Drug: Anti-PD-1 |
| 32 | NCT03452774 | SYNERGY-AI: Artificial Intelligence Based Precision Oncology Clinical Trial Matching and Registry | Recruiting | No Results Available | • Cancer, Metastatic • Cancer • Cancer of Pancreas • Cancer of Liver • Cancer of Stomach • Cancer Liver • Cancer of Rectum • Cancer of Kidney • Cancer of Esophagus • Cancer of Cervix • and 25 more | • Other: Clinical Trial Matching |
| 33 | NCT02876510 | ACTolog in Patients With Solid Cancers | Recruiting | No Results Available | • Cancer • Solid Tumor | • Drug: Fludarabine • Drug: Cyclophosphamide • Biological: IMA101 product • Biological: Recombinant human interleukin-2 • Diagnostic Test: IMA_Detect |
| 34 | NCT02366546 | Investigator Initiated Phase 1 Study of TBI-1301 | Recruiting | No Results Available | • Solid Tumors | • Drug: TBI-1301 • Drug: Cyclophosphamide • Drug: Fludarabine |
| 35 | NCT02096614 | Investigator Initiated Phase 1 Study of TBI-1201 | Recruiting | No Results Available | • Solid Tumors | • Drug: TBI-1201 • Drug: Cyclophosphamide • Drug: Fludarabine |
