# Supplementary material for: Ovarian cancer: Current status and strategies for improving therapeutic outcomes
Source: Cancer Med. 2019 Sep 27;8(16):7018–31. doi: 10.1002/cam4.2560 (PMC6853829; doi:10.1002/cam4.2560)
Supplement: Supplementary file 4 [file CAM4-8-7018-s004.docx]

**Table S4. Ongoing vaccine studies recruiting patients in Ovarian Cancer (Source: Clinical trial.gov)**

|  | **NCT Number** | **Title** | **Status** | **Indications** | **Interventions** |
| --- | --- | --- | --- | --- | --- |
| 1 | NCT00703105 | Ovarian Dendritic Cell Vaccine Trial | Recruiting | - Ovarian Cancer | - Biological: Ontak DC - Biological: DC vaccination - Drug: Ontak |
| 2 | NCT02432378 | Intensive Locoregional Chemoimmunotherapy for Recu rrent Ovarian Cancer Plus Intranodal DC Vaccines | Recruiting | - Cancer of Ovary - Cancer of the Ovary - Neoplasms, Ovarian - Ovarian Cancer - Ovary Cancer - Ovary Neoplasms | - Biological: Cisplatin + celecoxib + DC vaccine - Biological: Cisplatin + CKM + Celecoxib + DC Vaccine |
| 3 | NCT02978222 | Folate Receptor Alpha Peptide Vaccine With GM- CSF Versus GM-CSF  Alone in Patients With Platinum Sensitive Ovarian Can cer | Recruiting | - Platinum Sensitive Ovarian Cancer - Ovarian Cancer | - Biological: FR# peptide plus Adjuvant (GM-CSF) - Drug: Adjuvant (GM- CSF) Alone |
| 4 | NCT02933073 | Study of Oncoimmunome for the Treatment of Stage III  /IV Ovarian Carcinoma | Recruiting | - Ovarian Cancer | - Biological: OncoImmunome |
|  |  |  |  |  |  |
| 5 | NCT02785250 | Study of DPX-  Survivac Vaccine Therapy and Epacadostat in Patients With Recurrent Ovarian Cancer | Recruiting | - Recurrent Epithelial Ovarian Cancer - Recurrent Fallopian Tube Cancer - Recurrent Peritoneal Cancer | - Biological: DPX- Survivac - Drug: Cyclophosphamide - Drug: Epacadostat (INCB024360) |
| 6 | NCT03556566 | Open Label Immunotherapy Trial for Ovarian Cancer | Recruiting | - Ovarian Cancer | - Biological: Tableted vaccine (V3-OVA) containing ovarian cancer antigens |
| 7 | NCT03029611 | IGFBP-  2 Vaccine and Combination Chemotherapy in Treating Patients With Stage III-  IV Ovarian, Fallopian Tube, or Primary Peritoneal Canc er Undergoing Surgery | Recruiting | - Stage III Fallopian Tube Cancer - Stage III Ovarian Cancer - Stage III Primary Peritoneal Cancer - Stage IIIA Fallopian Tube Cancer - Stage IIIA Ovarian Cancer - Stage IIIA Primary Peritoneal Cancer - Stage IIIB Fallopian Tube Cancer - Stage IIIB Ovarian Cancer - Stage IIIB Primary Peritoneal Cancer - Stage IIIC Fallopian Tube Cancer - and 5 more | - Drug: Carboplatin - Procedure: Gynecological Surgical Procedure - Other: Laboratory Biomarker Analysis - Drug: Paclitaxel - Biological: pUMVC3- hIGFBP-2 Multi-Epitope Plasmid DNA Vaccine |
| 8 | NCT03029403 | Phase 2 Study of Pembrolizumab, DPX- Survivac Vaccine and  Cyclophosphamide in Advanced Ovarian, Primary Peritoneal or Fallopian Tube Cancer | Recruiting | - Advanced Cancer - Ovarian Cancer - Primary Peritoneal Carcinoma - Fallopian Tube Cancer | - Drug: Pembrolizumab - Biological: DPX- Survivac - Drug: Cyclophosphamide |
| 9 | NCT01376505 | Vaccine Therapy in Treating Patients With Metastatic Solid Tumors | Recruiting | - Malignant Solid Tumour - Breast Cancer - Malignant Tumor of Colon - GIST - Ovarian Cancer | - Biological: HER-2 vaccine - Biological: Extension HER-2 vaccine trial at OBD |
| 10 | NCT03300843 | Ability of a Dendritic Cell Vaccine to Immunize Melanoma or Epithelial  Cancer Patients Against Defined Mutated Neoantigens Expressed by the Autologous Cancer | Recruiting | - Melanoma - Gastrointestinal Cancer - Breast Cancer - Ovarian Cancer - Pancreatic Cancer | - Biological: Peptide loaded dendritic cell vaccine |
| 11 | NCT03162562 | The Safety and Antitumor Activity of the Combination of Oregovomab and  Hiltonol in Recurrent Advanced Ovarian Cancer | Recruiting | - Cancer of Ovary - Neoplasms, Ovarian - Ovarian Cancer Stage IV - Ovarian Cancer Recurrent - Ovarian Cancer Stage III - Ovary Cancer | - Biological: Oregovomab - Drug: Poly ICLC |
| 12 | NCT03100006 | Phase Ib/IIa Trial to Evaluate Oregovomab and Nivolu mab in Epithelial  Cancer of Ovarian, Tubal or Peritoneal Origin | Recruiting | - Epithelial Ovarian Cancer | - Drug: Nivolumab - Drug: Oregovomab |
|  |  |  |  |  |  |

| 13 | NCT02166905 | DEC-205/NY-ESO-1 Fusion Protein CDX- 1401, Poly ICLC, and IDO1  Inhibitor INCB024360 in Treating Patients With Ovarian  , Fallopian Tube,  or Primary Peritoneal Cancer in Remission | Recruiting | - Fallopian Tube Carcinoma - Ovarian Carcinoma - Primary Peritoneal Carcinoma |  | - Biological: DEC- 205/NY-ESO-1 Fusion Protein   CDX-1401   - Drug: Epacadostat - Other: Laboratory Biomarker Analysis - Other: Pharmacological Study - Drug: Poly ICLC |  |
| --- | --- | --- | --- | --- | --- | --- | --- |
| 14 | NCT00722228 | Autologous and Allogeneic Whole Cell Cancer Vaccine for Metastatic  Tumors | Recruiting | - Colorectal Cancer - Ovarian Cancer - Gastric Cancer - Breast Cancer - Lung Cancer - Kidney Cancer - Melanoma |  | - Biological: Autologous or Allogeneic tumor cells |  |
| 15 | NCT02498665 | A Study of DSP-  7888 Dosing Emulsion in Adult Patients With Advanced Malignancies | Recruiting | - Acute Myeloid Leukemia - Myelodysplastic Syndromes - Glioblastoma Multiforme - Melanoma - Non-Small Cell Lung Cancer - Ovarian Cancer - Pancreatic Cancer - Sarcoma - Renal Cell Carcinoma |  | - Drug: DSP-7888 Dosing Emulsion |  |

"
